# Genetic and Evolutionary Analyses of the Human Bone Morphogenetic Protein Receptor 2 (BMPR2) in the Pathophysiology of Obesity

**DOI:** 10.1371/journal.pone.0016155

**Published:** 2011-02-02

**Authors:** Dorit Schleinitz, Nora Klöting, Yvonne Böttcher, Sara Wolf, Kerstin Dietrich, Anke Tönjes, Jana Breitfeld, Beate Enigk, Jan Halbritter, Antje Körner, Michael R. Schön, Jost Jenkner, Yu-Hua Tseng, Tobias Lohmann, Miriam Dreβler, Michael Stumvoll, Matthias Blüher, Peter Kovacs

**Affiliations:** 1 Interdisciplinary Center for Clinical Research, University of Leipzig, Leipzig, Germany; 2 Department of Medicine, University of Leipzig, Leipzig, Germany; 3 Coordination Centre for Clinical Trials, University of Leipzig, Leipzig, Germany; 4 University Hospital for Children and Adolescents, University of Leipzig, Leipzig, Germany; 5 Municipal Clinic Karlsruhe, Clinic of Visceral Surgery, Karlsruhe, Germany; 6 Joslin Diabetes Center, Harvard Medical School, Boston, Massachusetts, United States of America; 7 Municipal Clinic, Dresden, Germany; University of Las Palmas de Gran Canaria, Spain

## Abstract

**Objective:**

Human bone morphogenetic protein receptor 2 (BMPR2) is essential for BMP signalling and may be involved in the regulation of adipogenesis. The *BMPR2* locus has been suggested as target of recent selection in human populations. We hypothesized that *BMPR2* might have a role in the pathophysiology of obesity.

**Research Design and Methods:**

Evolutionary analyses (dN/dS, Fst, iHS) were conducted in vertebrates and human populations. *BMPR2* mRNA expression was measured in 190 paired samples of visceral and subcutaneous adipose tissue. The gene was sequenced in 48 DNA samples. Nine representative single nucleotide polymorphisms (SNPs) were genotyped for subsequent association studies on quantitative traits related to obesity in 1830 German Caucasians. An independent cohort of 925 Sorbs was used for replication. Finally, relation of genotypes to mRNA in fat was examined.

**Results:**

The evolutionary analyses indicated signatures of selection on the *BMPR2* locus. *BMPR2* mRNA expression was significantly increased both in visceral and subcutaneous adipose tissue of 37 overweight (BMI>25 and <30 kg/m^2^) and 80 obese (BMI>30 kg/m^2^) compared with 44 lean subjects (BMI<25 kg/m^2^) (*P*<0.001). In a case-control study including lean and obese subjects, two intronic SNPs (rs6717924, rs13426118) were associated with obesity (adjusted *P*<0.05). Combined analyses including the initial cohort and the Sorbs confirmed a consistent effect for rs6717924 (combined *P* = 0.01) on obesity. Moreover, rs6717924 was associated with higher *BMPR2* mRNA expression in visceral adipose tissue.

**Conclusion:**

Combined *BMPR2* genotype-phenotype-mRNA expression data as well as evolutionary aspects suggest a role of *BMPR2* in the pathophysiology of obesity.

## Introduction

Despite recent advances achieved by genome wide association studies [1]–[3], the genetic factors contributing to the development of obesity are still elusive. The prevalence of obesity has increased particularly during the past 50 years. Since it is rather unlikely that human genotypes changed significantly during this period, it was probably the advent of Western lifestyle and unrestricted food availability that caused this dramatic increase. A popular theory to explain this observation is the “thrifty genotype” hypothesis [4] proposing that humans with a genetically increased capacity to store energy had an evolutionary advantage, since they were more likely to survive periods of food scarcity. In modern societies with easy access to high-caloric food and very little physical activity this former advantage is turned into its reverse and results in obesity. Although the hypothesis has been widely accepted, a clear approach to identify “thrifty” genes has not yet been postulated. However, the availability of high density single nucleotide polymorphism (SNP) genotyping arrays as well as rapidly emerging bioinformatics tools offer a possibility to reveal selective forces on loci dispersed throughout the genome. Employing these approaches, bone morphogenetic protein receptor 2 gene (*BMPR2*) was suggested as one of the potential targets of recent selection in European populations [5].

Bone morphogenetic proteins (BMPs) are members of the transforming growth factor-β (TGF-β) superfamily and are involved in the control of multiple key steps of embryonic development and differentiation [6]–[8]. BMPs are involved in adipocyte development, including adipose cell fate determination, differentiation of committed preadipocytes, and function of mature adipocytes [9]. The BMP signalling requires the formation of heteromeric complexes of BMP type 1 (BMPR1) and type 2 receptors (BMPR2) [10]–[12]. BMP binding either to a single receptor type subunit followed by the recruitment of the second subunit or to a pre-existing loose complex of both receptor types, activates two downstream signalling cascades: 1) SMAD proteins or 2) p38 mitogen activated protein kinase (p38MAPK) pathways [13]. Through their intracellular mediators (SMAD proteins) BMPs can trigger mesenchymal stem cells to enter the adipogenic and/or osteogenic lineage while preventing commitment into the myogenic lineage [14]. BMP2/4 induce white fat differentiation and BMP7 specify brown adipogenesis [15]. Since BMPR2 can bind both BMP2/BMP4 and BMP7 to activate the intracellular cascade involving SMAD intracellular signalling mediators [16], it might affect both the white and the brown adipogenesis. Also, over-expression of constitutively active BMP receptor 1A or 1B induces the commitment of C3H10T1/2 stem cells into adipocytes even in the absence of BMP2/4 [17]. We could recently show that *BMPR1A* mRNA expression in both visceral and subcutaneous adipose tissue as well as genetic variants in this gene strongly correlated with obesity and its related traits [18]. BMPR2, a serine threonine kinase located on chromosome 2q33-q34, is responsible for the trans-phosphorylation of BMPR1, further suggesting *BMPR2* as candidate gene for obesity.

Here, we initiated studies searching for signatures of selection for *BMPR2* in vertebrates and human populations. We analysed the coding region of *BMPR2* orthologs with the Phylogenetic Analysis by Maximum Likelihood (PAML). Furthermore, we used Haplotter/PhaseII database to screen for selection patterns within the *BMPR2* locus including intronic regions. Since *BMPR2* is also a plausible obesity candidate gene, we searched for evidence supporting the “thrifty gene” hypothesis by investigating its potential role in the pathophysiology of human obesity. We measured the expression of *BMPR2* mRNA in paired samples of human visceral and subcutaneous adipose tissue in subjects who had undergone detailed metabolic testing. To determine whether genetic variants in the *BMPR2* are related to adipose tissue *BMPR2* mRNA expression and to the obese human phenotype, we sequenced the *BMPR2* and evaluated the association between representative variants and obesity related traits in two independent Caucasian cohorts from Germany.

## Methods

### Study subjects

#### Ethics Statement

All studies were approved by the ethics committee of the University of Leipzig and all subjects gave written informed consent before taking part in the study.

#### Leipzig cohort

A total of 930 patients with type 2 diabetes (T2D) and 900 non-diabetic subjects recruited at the University Hospital in Leipzig (Germany) were included in the study. Healthy subjects included 288 males and 612 females (mean age 49±14 years; mean BMI 28.7±5.7 kg/m^2^; mean WHR 0.94±0.17; mean fasting plasma glucose 5.29±0.57 mmol/l; mean fasting plasma insulin 124±228 pmol/l). Patients with T2D included 475 males and 455 females (mean age 64±11 years; mean BMI 29.7±5.4 kg/m^2^) (data are given as arithmetic means±SD). In addition, oral glucose tolerance test (OGTT) and fasting plasma insulin measurements were performed in all non-diabetic subjects as previously described elsewhere [19]. In a subgroup of 374 non-diabetic subjects, insulin sensitivity was assessed with euglycemic-hyperinsulinemic clamps and plasma leptin and adiponectin measurements were carried out.

#### Tissue studies

Paired samples of visceral and subcutaneous adipose tissue were obtained from a subgroup of 190 Caucasian men (N = 91) and women (N = 99), who underwent open abdominal surgery (described in detail elsewhere) [19]. The age ranged from 16 to 99 years and body mass index from 21 to 55 kg/m^2^. In these subjects, in addition to above mentioned clinical parameters, abdominal visceral and subcutaneous fat area was calculated using computed tomography scans at the level of L4-L5 and percentage body fat was measured by dual-energy X-ray absorptiometry (DEXA).

#### Assays and measures of obesity and glucose metabolism

Fasting plasma insulin was measured with an enzyme immunometric assay for the IMMULITE automated analyser (Diagnostic Products Corporation, Los Angeles, CA, USA). Plasma leptin levels were assessed by radioimmunoassay (Linco Research, St. Charles, Mo, USA). The OGTT was performed after an overnight fast with 75 g standardized glucose solution (Glucodex Solution 75 g; Merieux, Montreal, Canada) and insulin sensitivity was assessed with the euglycemic-hyperinsulinemic clamp method as described elsewhere [20]; [21].

#### Sorbs cohort

The cohort was derived from the self-contained Sorbs population in Eastern Germany. The Sorbs represent a population of Slavonic origin who lived in ethnic isolation among the Germanic majority during the past 1,100 years. Today, the Sorbian-speaking, Catholic minority comprises approximately 15,000 full-blooded Sorbs resident in about ten villages in rural Upper Lusatia (Oberlausitz), Eastern Saxony [22]. Extensive phenotyping included standardised questionnaires for past medical history and family history, collection of anthropometric data (weight, height, WHR) and OGTT (diabetes definition according to the ADA criteria) [23]. Insulin was measured with the AutoDELFIA®Insulin assay (PerkinElmer Life and Analytical Sciences, Turku, Finland). Nine hundred twenty five Sorbs (740 subjects with normal glucose tolerance (NGT), 79 subjects with impaired glucose tolerance (IGT) and 106 cases with T2D; 542 females, 383 males) were available for the present study (mean age 48±16 years; mean BMI 27.0±4.9 kg/m^2^; mean WHR 0.87±0.10; mean fasting plasma glucose 5.54±1.18 mmol/l; mean fasting plasma insulin 40.7±27.4 pmol/l).

### Evolutionary analyses

We analysed patterns of evolution at the codon level across the *BMPR2* phylogeny (Figure S1A). The non-synonymous to synonymous substitution rate ratio (ω = dN/dS) of 36 *BMPR2* orthologs (http://www.ensembl.org, http://www.ncbi.nlm. nih.gov/gene; Figure S1A,B) was estimated by maximum likelihood methods [24] using the CODEML program (PAML4a package) [25]; [26]. In addition, phased (PHASE version 2.1) [27]; [28] SNP data of the HapMap populations (Utah residents with ancestry from northern and western Europe (CEU), Yoruba in Ibadan; Nigeria (YRI), Han Chinese in Beijing, China (CHB), Japanese in Tokyo, Japan (JPT)) were analysed for signatures of selection with DnaSP (version 5.00.03) [29], Sweep 1.8.1 and the Haplotter/PhaseII (http://hg-wen.uchicago.edu/selection/haplotter.htm). Provided are population genetic measures which comprises the fixation index (Fst) and standardized integrated haplotype score (iHS) [5].

### Analysis of human *BMPR2* mRNA expression

Human *BMPR2* expression was measured by quantitative real-time RT-PCR using SYBR Green methodology and fluorescence was detected on an ABI PRISM 7500 sequence detector (Applied Biosystems, Darmstadt, Germany) as described in detail elsewhere [19]. The following primers were used: human *BMPR2* (NCBI accession no NM_001204.5) 5′ CTTTACTGAGAATTTTCCACCTCCTG 3′ (sense) and 5′ GCCAAAGCAATGATTAT TGTCTCATC 3′ (antisense). Human *BMPR2* mRNA expression was calculated relative to the mRNA expression of *18S rRNA*, determined by a premixed assay on demand for human *18S rRNA* (PE Biosystems, Darmstadt, Germany). For 29 samples *BMPR2* cDNA amplification failed and therefore, only 161 were included in the study.

### Sequencing of the *BMPR2*

Sequencing of the *BMPR2* (13 exons, exon-intron boundaries; NM_001204 in the NCBI GenBank; 1271 bp in the 5′ region and 1282 bp in the 3′ UTR) in 48 non-related Caucasian subjects (12 lean subjects with NGT, 12 visceral obese, 12 subcutaneously obese, 12 with T2D) was performed using the Big Dye Terminator (Applied Biosystems, Inc., Foster City, CA) on an automated DNA capillary sequencer (ABI PRISM 3100 Avant; Applied Biosystems Inc., Foster City, CA). Sequence information and PCR conditions for all oligonucleotide primers used for variant screening are available upon request.

### Genotyping of *BMPR2* SNPs

SNP genotyping was done using the TaqMan SNP Genotyping assay according to the manufacturer's protocol (Applied Biosystems, Inc., Foster City, CA). To assess genotyping reproducibility, a random ∼5% selection of the sample was re-genotyped in all SNPs; all genotypes matched initial designated genotypes. 48 samples used for sequencing were also re-genotyped by TaqMan technique and perfectly matched the genotypes determined by sequencing.

### Statistical analyses

Prior to statistical analysis, non-normally distributed parameters were *ln*-transformed to approximate a normal distribution. Differences in genotype frequencies between the obese or diabetic cases and healthy controls were compared using logistic regression analyses. Multivariate linear relationships were assessed by generalized linear regression models. All analyses were done under the additive model and the presented *P*-values are adjusted for age, sex (and BMI for glucose traits). Differences in mRNA expression between visceral and subcutaneous adipose tissue were assessed using the paired Student's *t-*test.

Two-sided *P*-values <0.05 were considered to provide nominal evidence for association and are presented without correction for multiple hypothesis testing. The analysis of associations with quantitative traits was restricted to non-diabetics to avoid diabetes status or treatment masking potential effects of the variants on these phenotypic traits.

To obtain the combined effect of our two cohorts we performed a meta-analysis by using the METAL software [30]. The meta-analysis was performed in a fixed effects model by using the Mantel-Haenszel method. In haplotype analyses, groups of subjects carrying 2, 1, or 0 copies of the haplotype were compared. To estimate haplotypes the PHASE version 2.1 software was used [27]; [31]. The phase reconstruction method considers the unknown haplotypes as unobserved random quantities and aims to evaluate their conditional distribution in light of the genotype data.

Statistical analyses were performed using SPSS version 18 (SPSS, Inc.; Chicago, IL) and STATA (version 9.0), (StataCorp LP, Texas, USA).

## Results

### Evolutionary analyses

**Figure 1:**
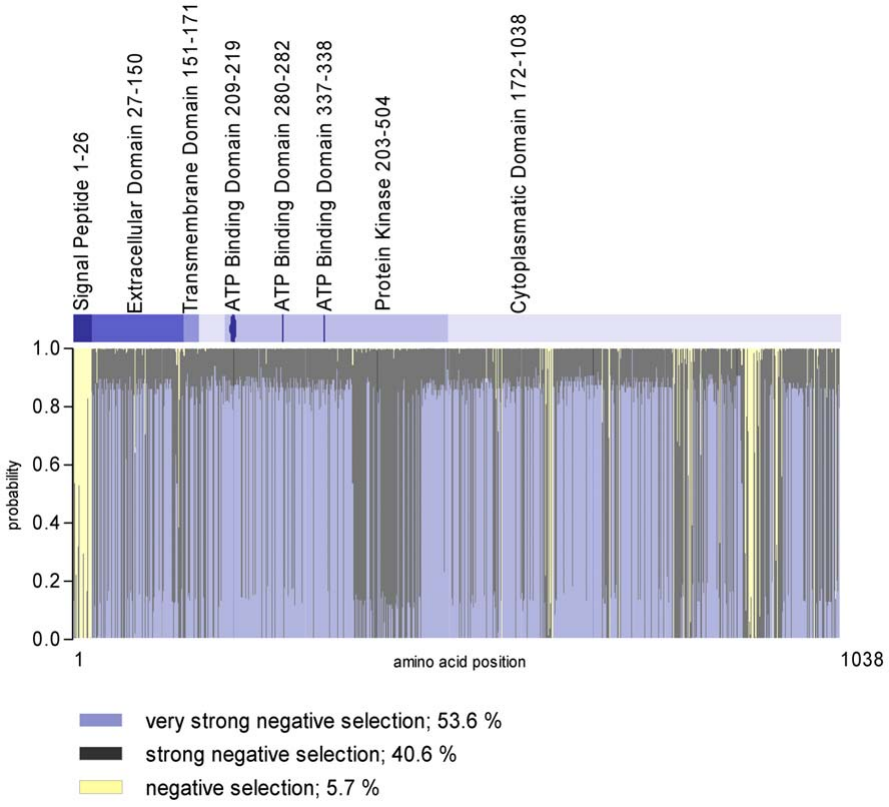
Site class probability bar plot for each codon across the *BMPR2* sequence indicating strength of conservation at each amino acid position. The degree of negative selection is equivalent to the strength of conservation. Included are 36 vertebrate orthologs. Human sequence was used as reference. Genetic domains above are annotated according to http://www.uniprot.org/uniprot/Q13873. Numbers represent the location of the domains (amino acids).

The dN/dS analyses revealed that the *BMPR2* is highly conserved among the 36 species analysed (average ω = 0.1278; Figure S1), hence being subjected to an overall purifying selection. Positional analyses indicate that 53.6% of positions are very strongly conserved, ω = 0.0037, 40.6% are strongly conserved, ω = 0.1247, and 5.7% are conserved, ω = 0.9974 (Figure 1). Except for one, none of the SNPs shows striking frequency differences between the observed populations as indicated by the fixation index Fst (Fst<0.2). For rs4675278, Fst-values indicate differences between European and East Asian populations (European *vs.* Chinese Fst = 0.2760, European *vs.* Japanese Fst = 0.3703), which is reflected in different allele frequencies and a change of minor allele: CEU: 0.267 (A-allele); CHB: 0.211 (G-allele); JPT: 0.136 (G-allele), YRI: 0.442 (A-allele).

The integrated haplotype score (iHS) is based on the differential levels of linkage disequilibrium surrounding a positively selected allele compared to the background allele at the same position (Table S1) [5]. For the European population iHS values below -2 were found for rs1980153 and rs16839127 (Table S1), i.e., haplotypes on the derived allele background are longer compared to ancestral allele background. In contrast, for rs6717924 the iHS was positive and nearly reached 2 in Caucasians (iHS = 1.986).

### Visceral and subcutaneous *BMPR2* mRNA expression and obesity

**Figure 2:**
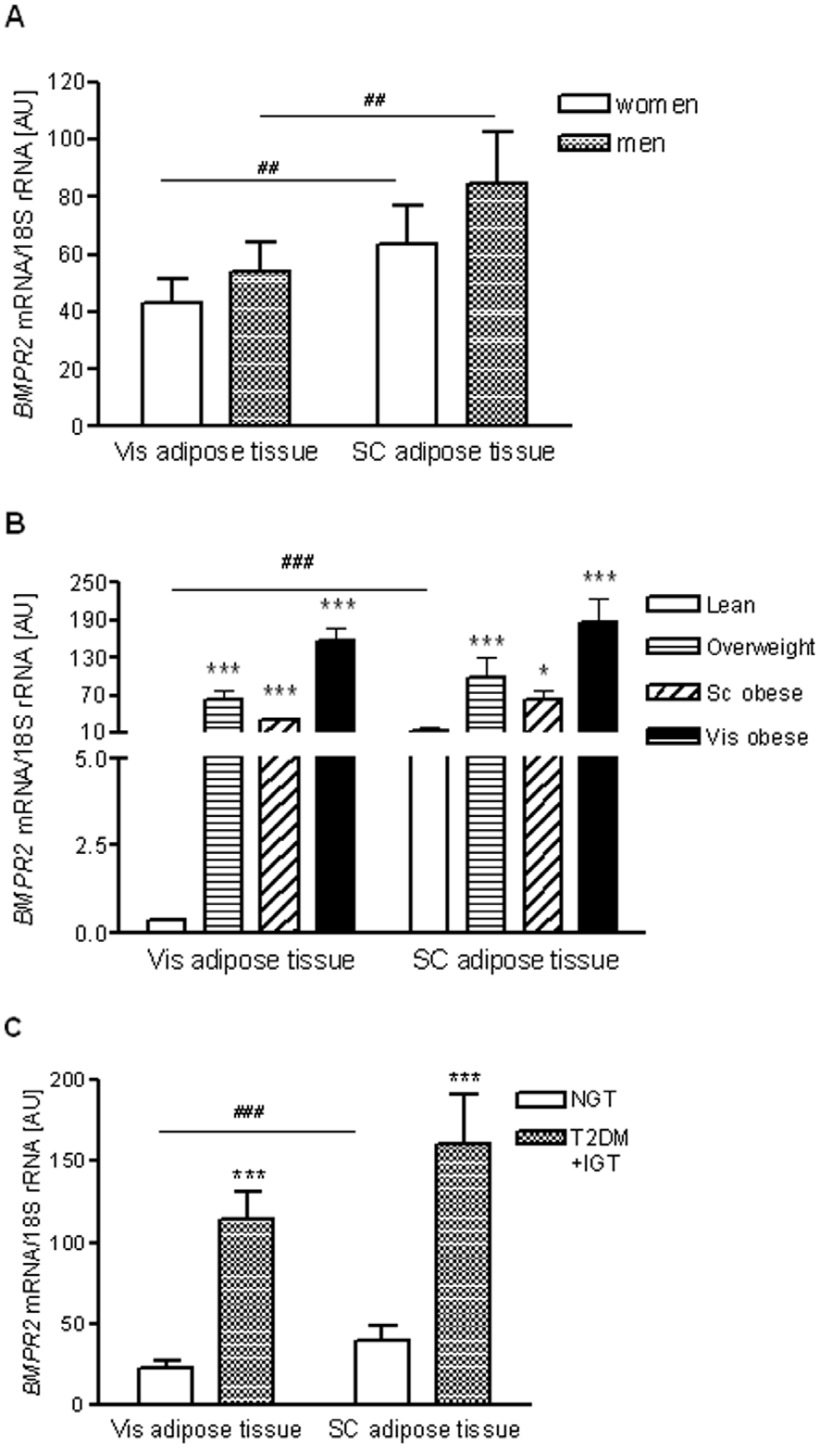
*BMPR2* mRNA expression in visceral (Vis) and subcutaneous (SC) adipose tissue in lean, obese and type 2 diabetic subjects. The data are means ±SEM; *BMPR2* mRNA levels in (A) men (N = 80) and women (N = 81), (B) subgroups of lean (BMI <25 kg/m^2^; N = 44), overweight (25 kg/m^2^ < BMI <30 kg/m^2^; N = 37), SC obese (N = 57) and Vis obese (N = 23) (BMI >30 kg/m^2^); (C) subjects with normal glucose tolerance (NGT; N = 116) and with impaired glucose tolerance (IGT; N = 15) or with type 2 diabetes (T2D; N = 30). * and *** indicate statistical significance at *P*<0.05 and *P*<0.001, respectively when compared with expression in lean subjects (Figure B) or in subjects with NGT (Figure C); # indicates statistical significance for differences between fat depots (Vis *vs.* SC) for appropriate subject groups; ^##^ and ^###^ indicate statistically significant at *P*<0.01 and *P*<0.001, respectively. AU, arbitrary units.

**Figure 3:**
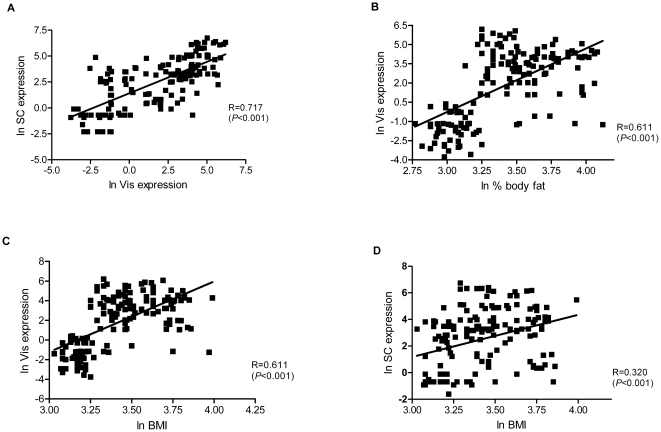
Correlations. A) between visceral (Vis) and subcutaneous (SC) *BMPR2* mRNA expression; B) between Vis *BMPR2* mRNA expression and % body fat, C) between Vis *BMPR2* mRNA expression and BMI, D) between SC *BMPR2* mRNA expression and BMI. Data are *ln*-transformed to achieve normal distribution.

Analysis of 161 paired samples of visceral and subcutaneous adipose tissue showed significantly higher *BMPR2* transcript levels in subcutaneous compared with visceral fat, independent of gender (Figure 2A). Even though the mRNA expression in men tended to be higher than in women, the gender differences did not reach statistical significance (Figure 2A). To investigate the expression according to body fat mass or fat distribution, we performed additional analyses in subgroups of lean (BMI<25 kg/m^2^), overweight (25 kg/m^2^<BMI<30 kg/m^2^), and obese (BMI>30 kg/m^2^) subjects. Based on CT scans measurement (L4-L5) of abdominal visceral and subcutaneous fat areas, obese subjects were further categorized as predominantly visceral (Vis) or subcutaneous (SC) obese as defined by a ratio of Vis/SC fat area >0.5 [19]. *BMPR2* mRNA expression was significantly increased in both visceral and subcutaneous adipose tissue of 37 overweight and 80 obese (57 subcutaneously obese and 23 visceral obese) compared with 44 lean subjects (*P*<0.001) (Figure 2B). Interestingly, the difference in *BMPR2* mRNA expression between visceral and subcutaneous adipose tissue was only seen in lean individuals, but not in overweight or obese subjects (Figure 2B). We further asked whether impaired glucose metabolism in patients with either IGT (N = 15) or T2D (N = 30) may be associated with altered *BMPR2* mRNA expression in different fat depots. Subgroup analyses demonstrated that IGT and T2D subjects were indistinguishable with regard to *BMPR2* mRNA expression; therefore these groups were analysed together. Patients with IGT/T2D had significantly higher *BMPR2* mRNA expression in both visceral and subcutaneous fat compared to NGT (N = 116) subjects (Figure 2C). We detected a significant correlation between visceral and subcutaneous *BMPR2* mRNA expression (Figure 3A).

### Correlation of *BMPR2* mRNA expression with parameters of obesity, glucose metabolism, and insulin sensitivity

Univariate regression analysis revealed significant positive correlations between both Vis and SC *BMPR2* mRNA expression and BMI, % body fat, waist, WHR, fasting and 2-hr plasma glucose, and fasting plasma insulin (Table 1; Figure 3). There was an inverse correlation between Vis and SC *BMPR1A* mRNA expression and glucose uptake during the steady state of a euglycemic-hyperinsulinemic clamp (Table 1). The correlations remained unchanged also after excluding subjects with IGT and T2D (data not shown). The correlation between *BMPR2* mRNA expression in fat and glucose infusion rate during the steady state of an euglycemic–hyperinsulinemic clamp remained significant even upon adjusting for age, gender and % body fat (Table 2).

**Table 1:** Linear regression analyses of visceral (Vis) and subcutaneous (SC) *BMPR2* mRNA expression with anthropometric and metabolic parameters (N = 161).

	Vis *BMPR2* mRNA	SC *BMPR2* mRNA
	R (*P*-value)	R (*P*-value)
**Age**	0.275 (<0.001)	0.183 (0.020)
**BMI**	0.611 (<0.001)	0.320 (<0.001)
**% body fat**	0.611 (<0.001)	0.321 (<0.001)
**WHR**	0.540 (<0.001)	0.322 (<0.001)
**Fasting plasma glucose**	0.303 (<0.001)	0.259 (0.001)
**Fasting plasma insulin**	0.777 (<0.001)	0.499 (<0.001)
**2-hr plasma glucose**	0.419 (<0.001)	0.361 (<0.001)
**GIR**	−0.815 (<0.001)	−0.603 (<0.001)

GIR – glucose infusion rate during the steady state of the euglycemic-hyperinsulinemic clamp.

**Table 2:** Multivariate linear regression analyses of visceral (Vis) and subcutaneous (SC) *BMPR2* mRNA expression with anthropometric and metabolic parameters (N = 161).

	Vis *BMPR2* mRNA	SC *BMPR2* mRNA
	β-Coefficient (*P*-value)	β-Coefficient (*P*-value)
**Model 1**		
**Age (years)**	0.049 (0.0004)	0.028 (0.021)
**Gender**	−0.010 (0.980)	0.030 (0.935)
**Model 2**		
**Age (years)**	0.025 (0.029)	0.017 (0.142)
**Gender**	0.010 (0.977)	0.032 (0.926)
**BMI**	6.792 (<0.001)	2.916 (<0.001)
**Model 3**		
**Age (years)**	0.030 (0.010)	0.019 (0.103)
**Gender**	−0.045 (0.893)	0.008 (0.981)
**% body fat**	4.710 (<0.001)	2.044 (<0.001)
**Model 4**		
**Age (years)**	0.011 (0.179)	0.005 (0.659)
**Gender**	−0.109 (0.663)	0.004 (0.990)
**% body fat**	2.325 (<0.001)	0.154 (0.771)
**GIR**	−2.898 (<0.001)	−2.047 (<0.001)

Model 1: correlation with age and gender as predictors of obesity;

Model 2: with BMI as measure of obesity and 3 with % fat as measure of obesity: test whether correlation between BMPR2 mRNA expression and obesity withstands adjustments for age and gender;

Model 4: test whether correlation between the BMPR2 mRNA expression and GIR is independent of obesity.

### Genetic variation in the *BMPR2*

**Figure 4:**
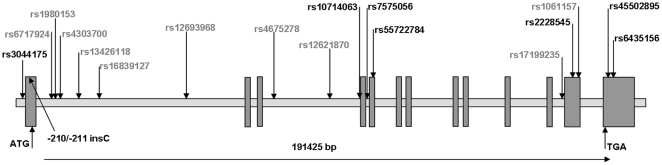
Genetic structure and SNPs analysed in the bone morphogenetic receptor 2 gene (*BMPR2*). The polymorphisms shown in black were discovered by direct gene sequencing. One of them was novel (w/o rs-number). Ten HapMap tagging polymorphisms (shown in grey) were analysed for association with obesity and related phenotypes in German subjects.

We sequenced the human *BMPR2* in 48 DNA-samples and found eight genetic variants (Figure 4). Further, ten HapMap tagging SNPs, covering 100% of the variation in the *BMPR2* locus (according to HapMap Phase II; www.hapmap.org) [32], were selected from the database using the Tagger software and the following criteria: minor allele frequency (MAF)>0.05 and r^2^>0.8. These SNPs were additionally genotyped in the 48 DNA-samples. We calculated the linkage disequilibrium (L.D.) between all SNPs using the EMLD statistical program (https://epi.mdanderson.org/qhuang/Software/pub.htm) (Table S2). Based on L.D., all SNPs identified through sequencing and with a minor allele MAF>0.05 were represented by at least one HapMap tagging SNP (L.D. with r^2^>0.8) (Table S2). Since two HapMap tagging SNPs were in 100% L.D. in our population (rs13426118 and rs1061157), only 9 tagging SNPs were therefore taken forward to genotyping for association studies in German Caucasians (Leipzig). For replication analyses we included the Sorbs from Germany. All SNPs were in Hardy-Weinberg equilibrium (*P*>0.05).

### BMPR2 variants and association with obesity

#### Leipzig cohort

In a case-control study including 447 lean (BMI<25 kg/m^2^) and 701 obese (BMI>30 kg/m^2^) subjects, rs6717924 and rs13426118 were nominally associated with obesity (*P*<0.05, adjusted for age and sex). Carriers of the minor allele for rs6717924 were at higher risk of being obese (odds ratio (OR) and [95% CI]  =  1.40 [1.06; 1.85], *P* = 0.018) whereas subjects with the minor allele for rs13426118 were protected against obesity (0.71 [0.55; 0.93], *P* = 0.013 in an additive mode of inheritance after adjusting for age and sex) (Table 3, Table S3).

**Table 3:** Association analyses of the *BMPR2* genetic variants with obesity in the Leipzig and the Sorbs cohorts.

			Leipzig (a) n = 1148			Sorbs (b) n = 552			Combined analysis	
	Major allele	Minor allele	Minor allele frequency (Case/Control)	OR [95% CI]	*P-value*	Minor allele frequency (Case/Control)	OR [95% CI]	*P-value*	OR [95% CI]	*P-value*
**rs6717924**	G	A	0.14/0.11	1.40 [1.06;1.85]	***0.018***	0.15/0.12	1.19 [0.77;1.84]	*0.43*	1.35 [1.07;1.70]	***0.011***
**rs13426118**	A	C	0.11/0.15	0.71 [0.55;0.93]	***0.013***	0.13/0.11	1.22 [0.78;1.91]	*0.39*	0.93 [0.72;1.21]	0.61

OR [95%CI]; odds ratio with 95% confidence intervals; *P-value*s were adjusted for age and sex.

*ORs indicate effect directions of the minor allele in additive mode of inheritance.

a) N = 701 cases (BMI>30 kg/m^2^) *vs.* 447 controls (BMI<25 kg/m^2^), b) N = 215 cases *vs.* 337 controls. Combined ORs and *P*-values represent the combined analysis of the two cohorts (Leipzig and Sorbs). The combined analyses were adjusted for age, sex and study cohort.

#### Sorbs

The two SNPs nominally associated with obesity in the Leipzig cohort were genotyped in the Sorbs for replication. None of the SNPs showed significant association in the Sorbs case-control study (337 lean and 215 obese subjects) (Table 3, Table S3).

In a combined analysis including both the Sorbs and the Leipzig cohort, only rs6717924 showed association with obesity (combined OR and CI- 1.35 [1.07; 1.70]; *P* = 0.011) (Table 3). The combined effects of the SNPs on obesity were confirmed in a meta-analysis using the metan command in STATA based on the estimated effect sizes of each study and their confidence intervals. The allele/genotype frequencies in the phenotypic groups (lean *vs.* obese) remained unchanged also after excluding subjects with T2D. However, due to smaller sample size, *P*-values did not reach statistical significance (data not shown).

### BMPR2 variants and association with obesity related quantitative traits

#### Leipzig cohort

Consistent with case-control studies, we found nominal associations between rs13426118 and BMI in 900 subjects without T2D. As expected, carriers of the obesity risk allele (major) had a higher mean BMI (Table 4) than the non-carriers.

**Table 4:** Association of *BMPR2* SNP variants with quantitative traits in the Leipzig (A) and the Sorbs (B) cohort.

Leipzig cohort (A)	BMI	WHR	Fasting plasma glucose	2-hr glucose OGTT	Fasting plasma insulin	GIR	% body fat	Leptin	Adiponectin
**rs6717924 G>A**									
***P*** **-value**	0.247	0.791	0.551	0.347	0.261	0.958	0.165	0.071	0.222
**β**	0.014	0.003	0.005	−0.019	−0.123	0.004	0.056	0.192	−0.099
**SE**	0.012	0.012	0.008	0.020	0.109	0.085	0.040	0.106	0.081
**rs1980153 A>T**									
***P*** **-value**	0.666	0.470	0.204	0.226	0.430	0.554	0.739	0.688	**0.019** *
**β**	−0.006	−0.010	−0.012	−0.028	0.104	0.057	−0.016	0.050	0.223
**SE**	0.014	0.014	0.009	0.023	0.132	0.097	0.047	0.124	0.094
**rs4303700 G>A**									
***P*** **-value**	0.206	0.743	0.470	0.768	0.103*	0.330	**0.021** *	0.976	0.691
**β**	−0.012	−0.003	−0.005	−0.005	0.140	−0.063	−0.075	0.003	−0.027
**SE**	0.010	0.010	0.007	0.016	0.086	0.065	0.032	0.085	0.067
**rs4675278 G>A**									
***P*** **-value**	0.077#	0.966	0.529	0.661	0.338	0.827	0.728	0.177*	0.572
**β**	0.013	−0.0003	0.003	−0.005	−0.061	0.011	0.008	−0.085	−0.027
**SE**	0.007	0.007	0.005	0.012	0.064	0.049	0.023	0.063	0.047
**rs16839127 G>A**									
***P*** **-value**	0.994	0.346	0.876	0.837	0.287#	0.823	0.641	0.639	0.742
**β**	−0.0001	0.015	0.002	0.005	0.146	0.022	−0.023	−0.062	0.033
**SE**	0.016	0.016	0.010	0.025	0.137	0.100	0.049	0.133	0.101
**rs12693968 G>A**									
***P*** **-value**	0.413	0.285	0.563	0.476	0.426	0.691	0.158	0.113	0.324
**β**	−0.008	−0.011	0.004	−0.012	−0.070	−0.027	0.045	0.134	−0.063
**SE**	0.010	0.010	0.007	0.016	0.087	0.068	0.032	0.085	0.064
**rs12621870 T>C**									
***P*** **-value**	0.827	0.572	0.280	0.203*	**0.025** *	0.350	0.559	0.539	0.195
**β**	0.002	−0.005	0.007	−0.019	−0.179	0.059	−0.017	−0.050	−0.078
**SE**	0.009	0.009	0.006	0.015	0.080	0.063	0.028	0.082	0.060
**rs17199235 A>G**									
***P*** **-value**	0.745	0.744	0.570	**0.042** *	0.058*	0.111	0.642	0.487	0.526
**β**	0.004	0.004	0.004	−0.037	−0.190	0.131	−0.017	0.074	−0.049
**SE**	0.011	0.011	0.008	0.018	0.100	0.082	0.037	0.106	0.077
**rs13426118 A>C**									
***P*** **-value**	0.016*	0.222	0.939	0.774	0.319	0.588	0.923	0.413#	0.597
**β**	−0.030	−0.015	−0.001	0.006	−0.107	−0.045	−0.004	0.089	−0.045
**SE**	0.012	0.012	0.008	0.020	0.107	0.083	0.042	0.109	0.085

Sorbian cohort (B)	BMI	WHR	Fasting plasma glucose	2-hrglucose OGTT	Fasting plasma insulin	2-hr insulin	p-Fat		
**rs6717924 G>A**									
***P*** **-value**	0.924	0.438	0.998	0.261	0.033*	0.030*	0.545		
**β**	0.001	−0.007	<0.001	−0.025	−0.084	−0.140	0.018		
**SE**	0.011	0.009	0.006	0.022	0.039	0.064	0.029		
**Combined ** ***P*** **-value**	0.366	0.731	0.665	0.145	**0.022**				
**rs13426118 A>C**									
***P*** **-value**	0.018*	0.406	0.790	0.616	0.061	0.443	0.096		
**β**	0.026	0.007	0.002	−0.011	−0.073	−0.049	0.047		
**SE**	0.011	0.008	0.006	0.022	0.039	0.064	0.028		
**Combined ** ***P*** **-value**	0.912	0.757	0.898	0.890	**0.044**				

The dataset included subjects with NGT and IGT (N = 900, except for % body fat, leptin and adiponectin (N = 374) for the Leipzig cohort; N = 819 for the Sorbs cohort). *P*-values were calculated after adjusting for age and gender for the variables BMI, WHR and % body fat; and for age, gender and BMI for the remaining variables. β indicate effect directions of the minor alleles. *P*-values are given for the additive model.

*/# significant in dominant/recessive mode of inheritance; dominant model indicates Mm+mm *vs.* MM; BMI = body mass index; WHR = waist-to-hip ratio; OGTT = oral glucose tolerance test; GIR = glucose infusion rate.

In addition, rs1980153 showed effects on adiponectin, rs4303700 on % body fat, rs12621870 on fasting plasma insulin, and rs17199235 on 2-hr plasma glucose levels (Table 4).

We also assessed the association of the nine SNPs with T2D in a case-control study including 930 cases with T2D and 722 healthy controls with NGT from the Leipzig cohort. No significant association with T2D was found under logistic regression analysis (*P*>0.05; adjusted for age, sex and BMI; data not shown).

#### Sorbs

The two SNPs (rs13426118, rs6717924) nominally associated with obesity in a case-control study in the Leipzig cohort, were also tested for associations with BMI and obesity related traits in 819 non-diabetic Sorbs. Although rs13426118 was nominally associated with BMI (*P*<0.05, adjusted for age and sex; Table 4), it showed opposite effect direction when compared with the Leipzig cohort. Moreover, rs6717924 was associated with fasting plasma insulin and 2-hr plasma insulin (*P*<0.05, adjusted for age, sex and BMI).

Combined *P*-values of the two *BMPR2* SNPs in our two study cohorts were assessed by a meta-analysis using the METAL software. Both SNPs, rs6717924 and rs13426118, showed nominal significant association with fasting plasma insulin (*P* = 0.022, and *P* = 0.044 respectively).

### Haplotype analyses in the Leipzig cohort

Using the PHASE version 2.1 software [27]; [31], we identified nine common haplotypes (frequency of each haplotype ≥0.05 among the ten *BMPR2* tagging SNPs. A summary of the haplotypes is given in Table S4. These nine haplotypes accounted for >80% of all observed haplotypes, where haplotypes were defined by the composition of alleles at each SNP in the following order: [rs6717924]-[rs1980153]-[rs4303700]-[rs13426118]-[rs16839127]-[rs12693968]-[rs4675278]-[rs12621870]-[rs17199235]-[rs1061157]. Three haplotypes were significantly associated with obesity. The frequency of the haplotypes [AAGAGAGTAG] and [GAGAGGATAG] were significantly increased in the obese group (21.0% vs. 14.0% and 15.9% vs. 10.8% in the lean subjects) whereas the haplotype [GAGCGAGTAA] showed increased frequency in lean controls (18.8% vs. 13.3% in the obese group) (*P* = 0.006, *P* = 0.006, and *P* = 0.021; adjusted for age and sex; heterozygote and homozygote haplotype carriers were grouped together due to small sample size in the homozygote haplotype carrier group). Furthermore, carriers of the haplotype [GAGAGGATAG] had significantly higher 2-hr plasma glucose levels and a lower glucose infusion rate compared to non-carriers (*P* = 0.011 and 0.026; adjusted for age, sex and BMI).

### Association of rs6717924 with *BMPR2* mRNA expression

**Figure 5:**
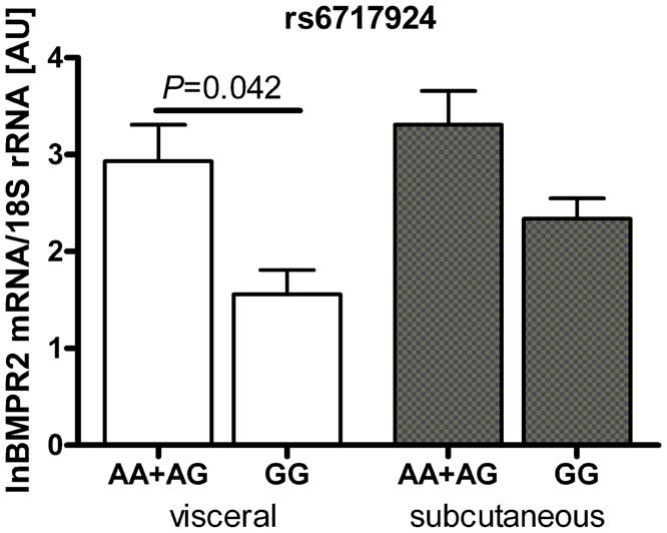
Association of rs6717924 with adipose tissue *BMPR2* mRNA expression. Given are means±SEM. A minor allele; G major allele.

Carriers of the rs6717924 A-allele (i.e. obesity risk allele) had higher *BMPR2* mRNA expression in visceral adipose tissue compared to carriers of the non-risk alleles (*P* = 0.042 adjusted for age, sex and BMI; Figure 5; N = 161). Consistent with the analyses in the entire cohort, the rs6717924 obesity risk allele was associated with BMI also in this subgroup (*P* = 0.017, adjusted for sex and age). Including visceral *BMPR2* mRNA expression as a covariate abolished the SNP effect on BMI (*P* = 0.31) while the effect on mRNA expression remained significant (*P*<0.001). None of the other SNPs was associated with either visceral or subcutaneous *BMPR2* mRNA expression.

## Discussion

BMPR2 plays an important role during embryonic development and remains ubiquitously expressed in later life. Employing genome wide screening strategies, the *BMPR2* locus has been suggested to be a target of recent evolutionary selection in human populations [5]. So far, genetic variants in the *BMPR2* have been described to be responsible for the majority of cases of the autosomal hereditable pulmonary arterial hypertension [33]. Functional studies revealed that vascular *BMPR2* mRNA expression is up-regulated in early stages of autoimmune diabetes in non-obese diabetic mice [34] and improved renal bone morphogenetic protein 7 (*BMP7*) and *BMPR2* mRNA expression following treatment in streptozotocin-induced diabetic rats is of benefit against renal damage during diabetic nephropathy [35]. However, little is known about the role of *BMPR2* in the pathophysiology of human obesity despite evidence for involvement of BMPs in the regulation of adipogenesis [9]. Also our recent studies on BMPR1A, partner in the receptor complex with BMPR2, suggest a regulatory role of these receptors in obesity [18]. We therefore performed a systematic study on the potential role of *BMPR2* in the pathophysiology of obesity by combining data from genetic, evolutionary, phenotypic and adipose tissue expression studies.

We analysed the coding region of 36 vertebrate *BMPR2* orthologs to screen for signatures of selections between species and showed that *BMPR2* has undergone an overall purifying selection. Considering strong physiologic relevance of *BMPR2* in developmental processes, it is not surprising that the coding region shows very strong conservation with more than half of amino acid positions being very strongly conserved. To assess intronic regions, we used Haplotter/PhaseII database to screen for selection patterns within the *BMPR2* locus. Population genetic measures represented by high iHS in Caucasian populations (Table S1) indicated signatures of recent selection at the *BMPR2* locus.

Since *BMPR2* is considered a plausible obesity candidate gene, we investigated its role in the pathophysiology of human obesity. We found significantly higher *BMPR2* mRNA expression in fat in overweight and obese compared to lean individuals. In addition, *BMPR2* mRNA expression was significantly higher in subjects with IGT/T2DM compared to controls with NGT. *BMPR2* mRNA expression correlated with measures of obesity and fat distribution, as well as with traits of glucose metabolism and insulin sensitivity. Notably, the visceral *BMPR2* expression increased remarkable in the overweight subjects. Furthermore, the difference in *BMPR2* mRNA expression between the two fat depots with higher expression in subcutaneous adipose tissue, predominantly in lean individuals, might suggest involvement of BMPR2 in the regulation of fat distribution. In such a case one would assume different expression patterns between both fat depots when comparing subcutaneously obese with viscerally obese patients. However, despite the highest expression levels (in both fat depots) found in viscerally obese patients, there were no depot differences in any of the “obese” groups (overweight, SC and Vis obese). Therefore, it seems that the depot differences observed in the total cohort were due to significant differences found in lean but not obese individuals. Correlations of *BMPR2* expression with obesity and relevant metabolic traits suggest that *BMPR2* mRNA expression in human adipose tissue might be related to progression of obesity but it remains unclear whether it is due to the extended fat mass or *vice versa*. Consistent with recent studies on *BMPR1A* [18], positive correlation of *BMPR2* mRNA expression with obesity suggests that obese individuals may have enhanced BMP-2/4 signalling, which may stimulate adipose tissue differentiation thereby contributing to increased fat mass. However, based on such correlation analyses, the direction of the causative chain can not be established and we can therefore not exclude that increased *BMPR2* expression may be a consequence rather than the cause of increased fat mass.

The genotype-phenotype association study showed two SNPs (rs6717924, rs13426118) nominally associated with obesity in the Leipzig cohort. However, they could not be replicated in the Sorbs, even though combined analyses of both cohorts supported the association of rs6717924 A-allele with obesity. Similarly, association of *BMPR2* SNPs with quantitative traits was moderate and would not withstand adjustment for multiple testing (e.g. Bonferroni correction). In a subgroup of 161 subjects the rs6717924 obesity risk allele was associated with visceral *BMPR2* mRNA expression and BMI. Including visceral *BMPR2* mRNA expression as a covariate abolished the SNP effect on BMI while the effect of mRNA expression levels remained significant. This suggests that the moderate SNP effect on BMI might be mediated through its effect on the visceral *BMPR2* mRNA expression level. Altered *BMPR2* mRNA expression might result in changes in adipose tissue expansion during the activation of division and differentiation of adipocyte precursor cells. Undoubtedly, studies targeting functional consequences of *BMPR2* obesity risk variants on *BMPR2* transcription are inevitable to clarify whether genetic variants can explain variation in *BMPR2* mRNA expression. Nevertheless, the iHS value of nearly 2 for the rs6717924 suggests that the haplotypes on the background of the ancestral allele A, which was associated with obesity and visceral mRNA levels, are longer compared to derived allele background and thus subject to recent positive selection.

Taking higher *BMPR2* mRNA levels in carriers of the rs6717924 A-allele into account, we used the Transcription Element Search System (TESS; http://www.cbil.upenn.edu/tess) to examine transcriptional binding sites surrounding this genetic variant, which might explain variation in *BMPR2* expression. The sequence surrounding rs6717924 (G>A) matches human transcription binding site for a transcription factor IPF1 (insulin upstream/promoter factor 1) for the G-allele. IPF1 is needed for the formation of the pancreas and is assumed to determine the fate of common pancreatic precursor cells and/or to regulate their propagation [36]. In this regard, it seems noteworthy that we observed nominal association of the rs6717924 with fasting plasma insulin levels in a combined analysis of both cohorts. Moreover, there is evidence that BMPs might regulate insulin secretion. BMP4 and its high-affinity receptor BMPR1A are expressed in differentiating and adult beta cells and transgenic expression of *Bmp4* in mice beta cells enhances glucose stimulated insulin secretion and glucose clearance [37]. Although *BMPR2* expression was higher in subjects with T2D, the genetic variation in *BMPR2* does not appear to be a major player in the polygenic aetiology of T2D as no association of genetic polymorphisms with T2D was found.

We are aware that statistical power of our study is limited due to relatively small sample size. In our cohorts we had a power of 80% (α = 0.05) to detect a difference of 0.71−1.32 kg/m^2^ per allele for BMI, 0.08−0.15 mmol/l for fasting plasma glucose, 3.94−18.07 pmol/l for fasting plasma insulin, 0.23−0.43 mmol/l for 2-hr glucose during an OGTT under the additive model. Similarly, in the case control studies for obesity, we had a power of 80% (α = 0.05) to detect SNP effects with odds ratios (ORs) ranging from 1.21 to 1.44 and from 1.35 to 1.37 in the Leipzig and Sorbs, respectively. Since the reported effect sizes for obesity SNPs are usually lower (ORs of 1.1–1.2), smaller effect may have been missed in our studies.

In conclusion, our data on expression, genetics as well as evolutionary aspects suggest a role of *BMPR2* in the pathophysiology of obesity and provide some evidence for thrifty genotype hypothesis.

## Supporting Information

Figure S1 **Phylogeny for** ***BMPR2*** **with estimates of ω for each species (A) and list of species analysed (B).** ω-values were obtained under the “free-ratio” model. C. = Common, N. =  Northern. * indicates species with concatenated sequences.
(TIF)

Table S1 **Integrated haplotype scores (iHS) for tagging SNPs within the** ***BMPR2*** **locus.** Provided are iHS values for Europeans (CEU), Africans (YRI) and Asians (ASN) (5).
(DOC)

Table S2 **Measures of L.D. (D prime (D′) and r^2^) among 17** ***BMPR2*** **variants.** r^2^ given in upper shaded boxes and D′ given in lower boxes. 1 = ′-918_-919insAGC, 2 = ′-210_-211insC, 3 = rs6717924, 4 = rs1980153, 5 = rs4303700, 6 = rs13426118, 7 = rs16839127, 8 = rs12693968, 9 = rs4675278, 10 = rs12621870, 11 = rs10714063, 12 = rs7575056, 13 = 137484 A/C, 14 = rs17199235, 15 = rs2228545, 16 = rs1061157, 17 = rs45502895.
(DOC)

Table S3 **Association of BMPR2 genetic variants with obesity in the Leipzig cohort (A) and the Sorbs cohort (B).** Case-control for obesity, 447 lean subjects (BMI<25 kg/m^2^) *vs.* 701 obese subjects (BMI>30 kg/m^2^). *P*-values were calculated after adjusting for age and gender. Odds ratios (OR) and 95% confidence intervals [95% CI] are given for the minor allele. Dominant model of inheritance indicates Mm+mm *vs.* MM. MAF = minor allele frequency.
(DOC)

Table S4 **Common haplotypes (frequency ≥5%) among the ten BMPR2 tagging SNPs.** Composition of alleles at each SNP in the following order: [rs6717924]-[rs1980153]-[rs4303700]-[rs13426118]-[rs16839127]-[rs12693968]-[rs4675278]-[rs12621870]-[rs17199235]-[rs1061157].
(DOC)
