# Supplementary figures and images for: Genetic and Evolutionary Analyses of the Human Bone Morphogenetic Protein Receptor 2 (BMPR2) in the Pathophysiology of Obesity
Source: PLoS One. 2011 Feb 2;6(2):e16155. doi: 10.1371/journal.pone.0016155 (PMC3032727; doi:10.1371/journal.pone.0016155)

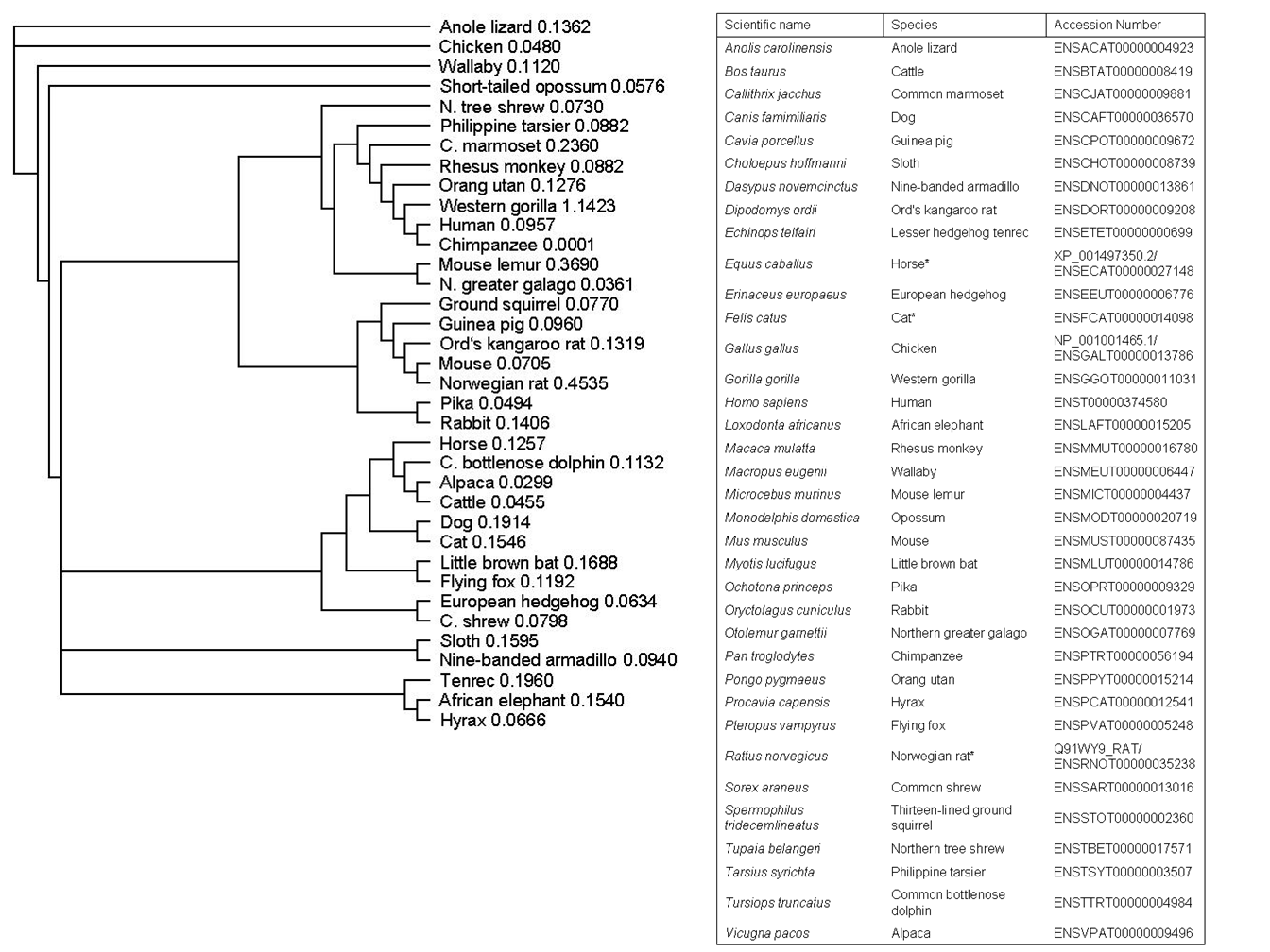

Supplement: Figure S1 — Phylogeny for BMPR2 with estimates of ω for each species (A) and list of species analysed (B). ω-values were obtained under the “free-ratio” model. C. = Common, N. = Northern. * indicates species with concatenated sequences. (TIF) [file pone.0016155.s001.tif]
