# Supplementary material for: Genetic and Evolutionary Analyses of the Human Bone Morphogenetic Protein Receptor 2 (BMPR2) in the Pathophysiology of Obesity
Source: PLoS One. 2011 Feb 2;6(2):e16155. doi: 10.1371/journal.pone.0016155 (PMC3032727; doi:10.1371/journal.pone.0016155)
Supplement: Table S1 — Integrated haplotype scores (iHS) for tagging SNPs within the BMPR2 locus. Provided are iHS values for Europeans (CEU), Africans (YRI) and Asians (ASN) (5). (DOC) [file pone.0016155.s002.doc]

**Table S1: Integrated haplotype scores (iHS) for tagging SNPs within the *BMPR2* locus.**

|  | **CEU** | **YRI** | **ASN** |
| --- | --- | --- | --- |
| **rs6177924** | **1.986** | **-1.498** | **-** |
| **rs1980153** | **-2.495** | **-** | **-** |
| **rs4303700** | **-1.667** | **-** | **-0.601** |
| **rs13426118** | **-1.711** | **-0.278** | **1.259** |
| **rs16839127** | **-2.236** | **-** | **-** |
| **rs12693968** | **1.235** | **-** | **-0.580** |
| **rs4675278** | **-0.572** | **-1.801** | **-1.864** |
| **rs12621870** | **-0.978** | **0.698** | **-1.971** |
| **rs17199235** | **-1.278** | **-** | **-** |
| **rs1061157** | **-1.485** | **-** | **0.981** |

**Provided are iHS values for Europeans (CEU), Africans (YRI) and Asians (ASN) [5].**
