# Supplementary material for: Genetic and Evolutionary Analyses of the Human Bone Morphogenetic Protein Receptor 2 (BMPR2) in the Pathophysiology of Obesity
Source: PLoS One. 2011 Feb 2;6(2):e16155. doi: 10.1371/journal.pone.0016155 (PMC3032727; doi:10.1371/journal.pone.0016155)
Supplement: Table S2 — Measures of L.D. (D prime (D′) and r2) among 17 BMPR2 variants. r2 given in upper shaded boxes and D′ given in lower boxes. 1 = ′-918_-919insAGC, 2 = ′-210_-211insC, 3 = rs6717924, 4 = rs1980153, 5 = rs4303700, 6 = rs13426118, 7 = rs16839127, 8 = rs12693968, 9 = rs4675278, 10 = rs12621870, 11 = rs10714063, 12 = rs7575056, 13 = 137484 A/C, 14 = rs17199235, 15 = rs2228545, 16 = rs1061157, 17 = rs45502895. (DOC) [file pone.0016155.s003.doc]

Table S2: Measures of L.D. (D prime (D’) and r^2^) among 17 *BMPR2* variants.

|  | 1 | 2 | 3 | 4 | 5 | **6** | **7** | 8 | 9 | 10 | 11 | 12 | 13 | 14 | 15 | 16 | 17 |
| --- | --- | --- | --- | --- | --- | --- | --- | --- | --- | --- | --- | --- | --- | --- | --- | --- | --- |
| 1 | 1 | 0.00 | 0.00 | 0.08 | 0.00 | 0.00 | 0.00 | 0.00 | 0.02 | 0.00 | 0.00 | 0.00 | 0.00 | 0.00 | 0.00 | 0.00 | 0.00 |
| 2 | 1.00 | 1 | 0.00 | 0.00 | 0.00 | 0.00 | 0.00 | 0.00 | 0.02 | 0.04 | 0.00 | 0.00 | 1.00 | 0.07 | 0.00 | 0.00 | 0.00 |
| **3** | 1.00 | 1.00 | 1 | 0.03 | 0.04 | 0.01 | 0.01 | 0.18 | 0.07 | 0.03 | **0.87** | **0.87** | 0.00 | 0.02 | 0.00 | 0.01 | 0.08 |
| 4 | 1.00 | 1.00 | 1.00 | 1 | 0.01 | 0.00 | 0.00 | 0.05 | 0.02 | 0.04 | 0.02 | 0.02 | 0.00 | 0.03 | 0.01 | 0.00 | 0.00 |
| 5 | 1.00 | 1.00 | 1.00 | 0.51 | 1 | 0.04 | 0.31 | 0.08 | 0.17 | 0.08 | 0.05 | 0.05 | 0.00 | 0.05 | 0.01 | 0.04 | 0.03 |
| **6** | 1.00 | 1.00 | 0.63 | 0.32 | 1.00 | 1 | 0.01 | 0.49 | 0.00 | 0.03 | 0.00 | 0.00 | 0.00 | 0.02 | 0.00 | **1.00** | 0.00 |
| 7 | 1.00 | 1.00 | 1.00 | 0.51 | 1.00 | 1.00 | 1 | 0.02 | 0.05 | 0.02 | 0.00 | 0.00 | 0.00 | 0.01 | 0.00 | 0.01 | 0.12 |
| 8 | 1.00 | 1.00 | 0.60 | 1.00 | 1.00 | 1.00 | 1.00 | 1 | 0.01 | 0.07 | 0.25 | 0.25 | 0.00 | 0.04 | 0.00 | 0.50 | 0.00 |
| 9 | 1.00 | 1.00 | 1.00 | 0.44 | 1.00 | 0.14 | 1.00 | 0.20 | 1 | 0.45 | 0.09 | 0.09 | 0.02 | 0.31 | 0.06 | 0.01 | 0.01 |
| 10 | 1.00 | 1.00 | 1.00 | 1.00 | 1.00 | 1.00 | 1.00 | 1.00 | 1.00 | 1 | 0.05 | 0.05 | 0.04 | 0.63 | 0.12 | 0.03 | 0.00 |
| 11 | 1.00 | 1.00 | 1.00 | 1.00 | 1.00 | 0.16 | 0.03 | 0.68 | 1.00 | 1.00 | 1 | **1.00** | 0.00 | 0.03 | 0.00 | 0.02 | 0.10 |
| 12 | 1.00 | 1.00 | 1.00 | 1.00 | 1.00 | 0.16 | 0.03 | 0.68 | 1.00 | 1.00 | 1.00 | 1 | 0.00 | 0.03 | 0.00 | 0.02 | 0.10 |
| 13 | 1.00 | 1.00 | 1.00 | 1.00 | 1.00 | 1.00 | 1.00 | 1.00 | 1.00 | 1.00 | 1.00 | 1.00 | 1 | 0.07 | 0.00 | 0.00 | 0.00 |
| **14** | 1.00 | 1.00 | 0.90 | 1.00 | 1.00 | 1.00 | 1.00 | 1.00 | 1.00 | 1.00 | 1.00 | 1.00 | 1.00 | 1 | 0.20 | 0.02 | 0.00 |
| 15 | 1.00 | 1.00 | 0.13 | 1.00 | 0.84 | 1.00 | 1.00 | 0.58 | 1.00 | 1.00 | 1.00 | 1.00 | 1.00 | 1.00 | 1 | 0.00 | 0.00 |
| 16 | 1.00 | 1.00 | 1.00 | 0.12 | 1.00 | 1.00 | 1.00 | 1.00 | 0.21 | 1.00 | 1.00 | 1.00 | 1.00 | 1.00 | 1.00 | 1 | 0.00 |
| 17 | 1.00 | 1.00 | 1.00 | 1.00 | 1.00 | 1.00 | 1.00 | 1.00 | 1.00 | 1.00 | 1.00 | 1.00 | 1.00 | 1.00 | 1.00 | 1.00 | 1 |

r^2^ given in upper shaded boxes and D’ given in lower boxes.

1**=´-918_-919insAGC,** 2**=´-210_-211insC,** 3**=rs6717924,** 4**=rs1980153,** 5**=rs4303700,** 6**=rs13426118,** 7**=rs16839127,** 8**=rs12693968,** 9**=rs4675278,** 10**=rs12621870,** 11**=rs10714063,** 12**=rs7575056,** 13**=137484 A/C,** 14**=rs17199235,** 15**=rs2228545,** 16**=rs1061157,** 17**=rs45502895.**
