# Supplementary material for: Genetic and Evolutionary Analyses of the Human Bone Morphogenetic Protein Receptor 2 (BMPR2) in the Pathophysiology of Obesity
Source: PLoS One. 2011 Feb 2;6(2):e16155. doi: 10.1371/journal.pone.0016155 (PMC3032727; doi:10.1371/journal.pone.0016155)
Supplement: Table S3 — Association of BMPR2 genetic variants with obesity in the Leipzig cohort (A) and the Sorbs cohort (B). Case-control for obesity, 447 lean subjects (BMI<25 kg/m2) vs. 701 obese subjects (BMI>30 kg/m2). P-values were calculated after adjusting for age and gender. Odds ratios (OR) and 95% confidence intervals [95% CI] are given for the minor allele. Dominant model of inheritance indicates Mm+mm vs. MM. MAF = minor allele frequency. (DOC) [file pone.0016155.s004.doc]

Table S3: Association of BMPR2 genetic variants with obesity in the Leipzig cohort (A) and the Sorbs cohort (B).

| **SNP** | **Genotype** | **Lean subjects (BMI<25g/m^2^)** | **Obese Subjects (BMI>30kg/m^2^)** | **MAF lean/obese** | **Additive *P*-value OR [95% CI]** | **Dominant *P*-value OR [95% CI]** | **Recessive *P*-value OR [95% CI]** |
| --- | --- | --- | --- | --- | --- | --- | --- |
| **Leipzig cohort (A)** | **Male/Female** | 183/264 | 257/444 |  |  |  |  |
|  | **Age (years)** | 50±18 | 59±11 |  |  |  |  |
|  | **BMI (kg/m^2^)** | 23.4±1.4 | 34.8±4.8 |  |  |  |  |
| **rs6717924** | **AA** | 7 (1.6%) | 11 (1.6%) |  | **0.018** | **0.011** | 0.854 |
|  | **AG** | 77 (18.0%) | 170 (25.1%) | 0.106/0.142 | 1.399 | 1.492 | 1.101 |
|  | **GG** | 344 (80.4%) | 496 (73.3%) |  | [1.059-1.848] | [1.098-2.027] | [0.397-3.051] |
| **rs1980153** | **TT** | 2 (0.5%) | 6 (0.9%) |  | 0.226 | 0.238 | 0.625 |
|  | **TA** | 65 (15.1%) | 111 (16.5%) | 0.080/0.091 | 1.222 | 1.231 | 1.506 |
|  | **AA** | 363 (84.4%) | 557 (82.6%) |  | [0.883-1.695] | [0.872-1.737] | [0.292-7.771] |
| **rs4303700** | **AA** | 17 (4.0%) | 30 (4.4%) |  | 0.236 | 0.203 | 0.776 |
|  | **AG** | 157 (36.3%) | 215 (31.7%) | 0.221/0.203 | 0.874 | 0.844 | 0.910 |
|  | **GG** | 258 (59.7%) | 433 (63.9%) |  | [0.700-1.092] | [0.651-1.095] | [0.476-1.739] |
| **rs4675278** | **AA** | 84 (19.4%) | 156 (23.0%) |  | 0.114 | 0.142 | 0.202 |
|  | **AG** | 122 (28.2%) | 192 (28.4%) | 0.335/0.372 | 1.138 | 1.209 | 1.225 |
|  | **GG** | 227 (52.4%) | 329 (48.6%) |  | [0.969-1.335] | [0.938-1.558] | [0.897-1.674] |
| **rs16839127** | **AA** | 0 (0%) | 3 (0.5%) |  | 0.560 | 0.674 |  |
|  | **AG** | 49 (11.4%) | 81 (12.0%) | 0.056/0.065 | 1.120 | 1.088 | - |
|  | **GG** | 381 (88.6%) | 590 (87.5%) |  | [0.765-1.639] | [0.735-1.610] |  |
| **rs12693968** | **AA** | 30 (7.4%) | 39 (5.9%) |  | 0.765 | 0.443 | 0.440 |
|  | **AG** | 124 (30.5%) | 225 (34.3%) | 0.227/0.231 | 1.033 | 1.110 | 0.815 |
|  | **GG** | 252 (62.1%) | 392 (59.8%) |  | [0.835-1.277] | [0.850-1.450] | [0.484-1.370] |
| **rs12621870** | **CC** | 36 (8.3%) | 47 (6.9%) |  | 0.461 | 0.739 | 0.250 |
|  | **CT** | 153 (35.4%) | 243 (35.7%) | 0.260/0.248 | 0.928 | 0.958 | 0.756 |
|  | **TT** | 243 (56.3%) | 390 (57.4%) |  | [0.759-1.133] | [0.742-1.236] | [0.469-1.218] |
| **rs17199235** | **GG** | 12 (2.8%) | 16 (2.4%) |  | 0.941 | 0.999 | 0.813 |
|  | **GA** | 98 (23.3%) | 163 (24.1%) | 0.145/0.144 | 0.990 | 1.000 | 0.907 |
|  | **AA** | 312 (73.9%) | 497 (73.5%) |  | [0.769-1.276] | [0.747-1.338] | [0.403-2.039] |
| **rs13426118** | **CC** | 10 (2.3%) | 7 (1.0%) |  | **0.013** | **0.022** | 0.139 |
|  | **CA** | 108 (24.9%) | 136 (20.0%) | 0.147/0.110 | 0.714 | 0.708 | 0.467 |
|  | **AA** | 316 (72.8%) | 537 (79.0%) |  | [0.546-0.932] | [0.527-0.951] | [0.170-1.281] |
| **Sorbian cohort (B)** | **Male/Female** | 104/233 | 78/137 |  |  |  |  |
|  | **Age (years)** | 39±15 | 58±12 |  |  |  |  |
|  | **BMI (kg/m^2^)** | 22.3±1.7 | 33.9±3.6 |  |  |  |  |
| **rs6717924** | **AA** | 2 (0.6%) | 3 (1.4%) |  | 0.425 | 0.498 | 0.476 |
|  | **AG** | 78 (23.5%) | 55 (26.2%) | 0.123/0.145 | 1.192 | 1.174 | 2.035 |
|  | **GG** | 252 (75.9%) | 152 (72.4%) |  | [0.774-1.838] | 0.739-1.864] | [0.289-14.342] |
| **rs13426118** | **CC** | 3 (1.0%) | 2 (1.0%) |  | 0.386 | 0.267 | 0.517 |
|  | **CA** | 67 (20.7%) | 51 (24.4%) | 0.113/0.132 | 1.219 | 1.315 | 0.533 |
|  | **AA** | 253 (78.3%) | 156 (74.6%) |  | [0.779-1.909] | [0.811-2.131] | [0.080-3.574] |

Case-control for obesity: (A) 447 lean subjects (BMI<25kg/m^2^) *vs.* 701 obese subjects (BMI>30kg/m^2^); (B) 337 lean subjects (BMI<25kg/m^2^) *vs.* 215 obese subjects (BMI>30kg/m^2^). *P*-values were calculated after adjusting for age and gender. Odds ratios (OR) and 95 % confidence intervals [95 % CI] are given for the minor allele. Dominant model of inheritance indicates Mm+mm *vs.* MM. MAF=minor allele frequency.
