# Supplementary material for: Genetic and Evolutionary Analyses of the Human Bone Morphogenetic Protein Receptor 2 (BMPR2) in the Pathophysiology of Obesity
Source: PLoS One. 2011 Feb 2;6(2):e16155. doi: 10.1371/journal.pone.0016155 (PMC3032727; doi:10.1371/journal.pone.0016155)
Supplement: Table S4 — Common haplotypes (frequency ≥5%) among the ten BMPR2 tagging SNPs. Composition of alleles at each SNP in the following order: [rs6717924]-[rs1980153]-[rs4303700]-[rs13426118]-[rs16839127]-[rs12693968]-[rs4675278]-[rs12621870]-[rs17199235]-[rs1061157]. (DOC) [file pone.0016155.s005.doc]

**Table S4: Common haplotypes (frequency ≥5%) among the ten *BMPR2* tagging SNPs.**

| ***BMPR2* haplotype** | **Frequency** |
| --- | --- |
| [GAAAGGGTAG] | 14.3 % |
| [GAGAGGGTAG] | 13.3 % |
| [GAGAGGACGG] | 12.9 % |
| [GAGAGGACAG] | 9.9 % |
| [AAGAGAGTAG] | 9.9 % |
| [GTGAGGGTAG] | 7.8 % |
| [GAGCGAGTAA] | 7.6 % |
| [GAGAGGATAG] | 6.9 % |
| [GAAAAGGTAG] | 6.1 % |

Composition of alleles at each SNP in the following order: [rs6717924]-[rs1980153]-[rs4303700]-[rs13426118]-[rs16839127]-[rs12693968]-[rs4675278]-[rs12621870]-[rs17199235]-[rs1061157].
